# Potentials of typical plant species from rewetted fenlands for the supply of strategic elements

**DOI:** 10.1038/s41598-025-05180-0

**Published:** 2025-06-20

**Authors:** Karina Michalska, Monika Heiermann, Carsten Lühr, Björn Meermann, Ralf Pecenka, Andreas Schulz, Nicole Langhammer, Susanne Theuerl, Annette Prochnow

**Affiliations:** 1 Leibniz Institute for Agricultural Engineering and Bioeconomy (ATB), Max-Eyth-Allee 100, 14469 Potsdam, Germany; 2 Albrecht Daniel Thaer Institute for Agricultural and Horticultural Sciences, Faculty of Life Sciences, Humboldt-University of Berlin, Hannoversche Str. 27, 10115 Berlin, Germany; 3 Division 1.1 – Inorganic Trace Analysis, Federal Institute for Materials Research and Testing (BAM), Richard-Willstätter-Straße 11, 12489 Berlin, Germany

**Keywords:** Peatlands, Paludiculture, Ge, Si, Rare earth elements, Bioeconomy, Element cycles, Environmental sciences, Ecology, Wetlands ecology

## Abstract

Rewetting of peatlands requires the development of new biomass utilization pathways. The supply of strategic elements with key importance for the development of priority technologies, such as germanium (Ge), silicon (Si) and rare earth elements, from fenland plants is one option. To provide a first estimation of the potential, concentrations of strategic elements were determined in nine biomass samples covering typical fenland vegetation in northeast Germany. Subsequently, a simplified estimation of potential revenue from strategic element recovery was made. The analysed plant species can be classified as high or intermediate Si plant accumulators with highest contents of more than 16.0 g Si kg^−1^ dry mass (DM) in sedges and common reeds. Ge concentrations were lower with reed canary grass containing the highest amounts of 465.3 µg Ge kg^−1^ DM. Simultaneous acquisition of Ge and Si could provide higher total element yields and revenues of up to 500 $ ha^−1^. In contrast, the potentials for supplying rare earth elements appeared to be very low, with common reed containing the highest sum of rare earth elements of 437.4 µg kg^−1^ DM. Biomass from rewetted fenlands is capable of accumulating strategic elements. More knowledge is required to understand the factors affecting their accumulation.

**Supplementary Information:**

The online version contains supplementary material available at 10.1038/s41598-025-05180-0.

## Introduction

Peatlands are formed by the long-term accumulation of organic matter made of slowly decaying biomass under exclusion of air due to high water levels^1–3^. Covering about 3% of the global land area^4^, peatlands store an estimated 21 to 44% of the carbon sequestered in soils^5^ and large amounts of nutrients^6^. Peatlands are also known to provide various ecosystem services, playing a crucial role in water regulation, and maintaining biodiversity^2,7,8^.

In Germany, organic soils cover an area of 1.82 million ha^9^. Their predominant use is agriculture. Large-scale deep drainage of organic soils in the 1960s to 1980s has caused wide-ranging degradation of peatlands^3^ and severe detrimental impacts on their ecological functions. Drained organic soils turn from sinks to sources of carbon and nitrogen, thus contributing a considerable share of 6.6% to the national greenhouse gas emissions^9^ and impairing water quality^7,10^. Degraded peatlands are less capable of holding water in the landscape^11,12^ and harbouring specialized plant and animal species^2,7,10,13^.

Rewetting of peatlands is regarded as one of the most effective measures to mitigate greenhouse gas emissions from land use, land use change^14–17^ and to restore the water retention function in the watershed^18,19^. In topogenic fenlands, i.e. those that would form due to the topography of the landscape, as they prevail in the northeast federal states of Germany, raising water tables induces the establishment of tall, graminoid wetland plants^20^ such as reed canary grass (*Phalaris arundinacea*), broad-leafed cattail (*Typha latifolia*), common reed (*Phragmites australis*), tall sedges (*Carex* spec*.*) and rushes (*Juncus* spec*.*)^20–22^. Thus, rewetted peatlands are no longer available for use as grasslands or arable land. New management strategies for wet peatlands referred to as paludiculture are needed^10,22^, with the derived plant biomass referred to as paludibiomass. While a number of paludibiomass-based products such as paper and packaging^23^, insulation and construction materials^7,10^ and substrates for horticulture and/or peat substitute^10,24^ are currently at the stage of advanced research, prototyping and even implementation, alternative solutions are still being sought. The supply of strategic elements from paludibiomass might be one option.

In 2024, the European Commission established the Critical Raw Materials Act^25^ which covers 34 raw materials of key importance for the development of priority technologies. 17 of those materials are considered strategic^25^. Strategic elements (SEs) include, among others, germanium (Ge), silicon (Si) and 7 of the rare earth elements (REEs). Rare earth elements are usually referred to as a group of 17 chemically similar metallic elements comprising the 15 lanthanide elements (lanthanum (La), cerium (Ce), praseodymium (Pr), neodymium (Nd), promethium (Pm), samarium (Sm), europium (Eu), gadolinium (Gd), terbium (Tb), dysprosium (Dy), holmium (Ho), erbium (Er), thulium (Tm), ytterbium (Yb), lutetium (Lu)), together with yttrium (Y) and scandium (Sc)^26^. Commonly they are divided into light (LREEs) and heavy (HREEs) rare earth elements. In this publication, the term “strategic elements” refers to all REEs, Ge and Si.

Strategic elements are essential raw materials in existing and emerging high-technology applications in the fields of transport, information, communication, energy, chemistry and medicine^27^. They are used for the production of e.g. semiconductor wafers, computers, mobile phones, rechargeable batteries, solar cells and wind turbines^28,29^ and can be found in catalysts, polishing compounds and alloys^30^. The demand for SEs increases rapidly, reflecting the growth of the high-tech sectors mentioned above. Already in 2009 the estimated global demand for REEs grew by 10 to 20% per annum^31^. By 2040, demand for REEs is expected to reach 3–7 times its current level, while supply is expected to only double^32^.

The majority of REEs are nowadays acquired through mining the Earth’s crust and deposits while Ge so far is mainly obtained from either fly ashes or coproduction during silver, copper and zinc production^33,34^. Long and complicated extraction and purification procedures require many different chemicals and reagents, which may result in negative impacts on the environment^35–37^ and subsequently pose a threat also to human health^38,39^. Due to concentration of production in a small number of countries with China accounting for approximately 95% of the total production, the market is highly susceptible to strong price fluctuations and disruptions^40^. Recycling and reuse strategies are limited by the very low recovery level of REEs of only about 1% of the final product^41^. Therefore, the search for alternative resources of SEs has become a critical issue.

Soils and biota may represent more environmentally friendly options for acquiring SEs. The concentrations of strategic elements in soils vary depending on the parent material and the contents of clay, carbonate, organic matter, aluminium, iron, and manganese^42–45^, with typical concentrations in soils ranging from 16 to 700 mg kg^−1^ for sum of REEs^46^, 2 mg kg^−1^ for Ge^44^ and from below 0.1 up to 60 g kg^−1^ for amorphous Si^47–50^. The organic matter in peatland soils originates from plants, which are known to contain relatively low amounts of rare earth elements. This is assumed to explain the low contents of REEs found in two organic soils in northeast Germany (sum REEs 13.3 and 15.9 mg kg^−1^ dry mass (DM)^51^). Positively charged REEs are however adsorbed, chelated or complexed by organic matter’s negatively charged groups^46^. Therefore, it is generally believed that REEs tend to bind to the organic matter. Their ultimate concentrations can vary significantly depending on redox conditions, which in the case of peatlands are directly dictated by the water table^52^. In the peat itself, the occurrence of SEs has not been extensively studied so far.

Plants take up strategic elements from the soil solution in the form of ions and sometimes as a soluble complex^42,44,53^. It is generally assumed that SEs are absorbed by plant roots through the rhizospheric soil solution containing dissolved SEs^54,55^. After having been taken up, SEs are transferred into the xylem vessels and transported to the aerial parts of the plant. This transport is mediated by water flow and driven by the transpiration stream^56^. After reaching the stems and leaves SEs precipitate as a result of oversaturation^36,57,58^. Both the uptake and transport mechanisms of REEs in plants are not selective and do not differ from mechanisms used for transporting other elements, regardless of the plants physiology^36,59^. On the other hand, a number of studies claim that Ge as a chemical analogue of Si is accumulated in plants unintentionally during acquisition of the latter by means of shared passive and active uptake mechanisms mediated by various influx- and efflux transporters^44,60–62^. The Si precipitates, commonly known as phytoliths^63^, contain not only Ge, but also REEs and a wide range of metal(loids) such as Zn, As, Cd or Cu^56,64^. Concentrations of SEs have been investigated for several cultivated plants such as maize, rice and wheat, showing wide variation between species and plant compartments^65–67^. Like other plants, biomass from paludiculture may be considered as a potential source for SEs. Biomass yields from self-establishing vegetation on rewetted fenland sites can usually be expected to be high^21^. However, to the best of our knowledge there is no data on concentrations of strategic elements in paludibiomass so far.

The objective of this study is to provide first insights on concentrations of chosen strategic elements in paludibiomass and to estimate potential element yields and revenues from typical fenland vegetation. Biomass samples were taken from various rewetted fenland fields in northeast Germany and analysed for their concentrations of SEs. Based on biomass yields from literature and actual market prices, potential element yields and revenues were calculated. Options of integrating SE supply from paludibiomass into sustainable bioeconomy strategies are discussed.

## Results

### Concentrations of strategic elements in fenland vegetation

SE concentrations in the biomass samples differed among plant species and site of origin (Table 1). For REEs analysis more than 53% of results were below their respective limits of quantification (LoQ). Therefore, they were excluded from the calculation of the total amount of REEs for each individual sample. Similarly, for Ge concentrations 44% of the results did not exceed LoQ and were not counted in further evaluation. All the obtained results from Si analysis were higher than LoQ.

**Table 1:** Concentrations of individual strategic elements in tested fenland plant samples (< LoQ—below quantification limit; measure of variability ( ±) given as standard deviation for 2 (^b^) or 3 (^c^) repetitions, single measurement denoted as (^a^).

Element	Content (µg kg^−1^ DM)									
	Sample	Common reed			Broad-leafed cattail		Reed canary grass	Sedges	Rushes	Mixed vegetation
	Number	#1	#2	#3	#4	#5	#6	#7	#8	#9
	LoQ									
La	339.4	< LoQ	555.4^a^	< LoQ	< LoQ	< LoQ	< LoQ	< LoQ	< LoQ	< LoQ
Ce	286.5	< LoQ	478.6^a^	< LoQ	< LoQ	< LoQ	< LoQ	< LoQ	< LoQ	< LoQ
Pr	2.0	2.7 ± 0.5^c^	14.3 ± 10.7^c^	2.6 ± 0.3^c^	14.5 ± 0.6^c^	2.6 ± 0.4^c^	4.4 ± 1.9^c^	4.8 ± 0.8^c^	4.3 ± 0.8^c^	3.2 ± 1.0^c^
Nd	3.4	11.3 ± 1.6^c^	54.6 ± 39.7^c^	9.7 ± 1.8^c^	53.8 ± 2.9^c^	10.3 ± 1.0^c^	16.6 ± 7.1^c^	17.6 ± 3.1^c^	16.8 ± 2.7^c^	12.4 ± 3.0^c^
Sm	2.3	< LoQ	9.1 ± 6.7^c^	< LoQ	9.5 ± 0.4^c^	< LoQ	3.9^a^	3.2 ± 0.7^c^	2.9 ± 0.6^c^	2.5^a^
Eu	3.3	< LoQ	13.2 ± 5.3^c^	7.6 ± 2.7^c^	7.1 ± 0.2^c^	6.6 ± 0.6^c^	17.6 ± 0.7^c^	13.8 ± 0.3^c^	9.4 ± 0.9^c^	6.4 ± 0.5^c^
∑ LREE		14.0 ± 1.7	1125.2 ± 42.0	19.9 ± 3.2	84.9 ± 3.0	19.5 ± 1.2	42.5 ± 7.4	39.3 ± 3.3	33.4 ± 3.0	24.5 ± 3.1
Gd	2.4	3.1 ± 0.1^c^	14.1 ± 10.6^c^	22.5 ± 34.0^c^	13.8 ± 1.0^c^	3.0 ± 0.3^b^	4.9 ± 2.0^c^	5.1 ± 0.6^c^	4.3 ± 0.5^c^	9.8 ± 12.1^c^
Tb	1.7	< LoQ	3.5^a^	< LoQ	2.2 ± 0.4^c^	< LoQ	< LoQ	< LoQ	< LoQ	< LoQ
Dy	2.3	< LoQ	5.8 ± 3.0^c^	< LoQ	8.1 ± 2.1^c^	< LoQ	< LoQ	2.6 ± 0.3^b^	< LoQ	< LoQ
Ho	1.8	< LoQ	< LoQ	< LoQ	2.0^a^	< LoQ	< LoQ	< LoQ	< LoQ	< LoQ
Er	1.8	1.9^a^	4.4 ± 2.0^c^	< LoQ	6.2 ± 1.4^c^	< LoQ	1.9^a^	2.1 ± 0.1^b^	< LoQ	< LoQ
Tm	1.8	< LoQ	< LoQ	< LoQ	< LoQ	< LoQ	< LoQ	< LoQ	< LoQ	< LoQ
Yb	2.0	< LoQ	3.5 ± 1.0^b^	< LoQ	4.4 ± 1.2^c^	< LoQ	< LoQ	< LoQ	< LoQ	< LoQ
Lu	2.4	< LoQ	< LoQ	< LoQ	< LoQ	< LoQ	< LoQ	< LoQ	< LoQ	< LoQ
Sc	49.6	54.7^a^	< LoQ	< LoQ	< LoQ	< LoQ	< LoQ	< LoQ	< LoQ	< LoQ
Y	1.8	10.2 ± 5.6^c^	20.2 ± 4.2^c^	9.2 ± 2.1^c^	48.5 ± 15.8^c^	8.4 ± 1.6^c^	6.6 ± 1.2^c^	16.0 ± 1.3^c^	12.3 ± 2.0^c^	9.3 ± 1.5^c^
∑ HREE		69.9 ± 5.6	51.4 ± 12.0	31.7 ± 34.1	85.1 ± 16.1	11.4 ± 1.6	13.3 ± 2.4	25.8 ± 1.5	16.6 ± 2.0	19.0 ± 12.2
∑ REE		84.0 ± 5.9	1176.6 ± 43.7	51.5 ± 34.3	170.0 ± 16.4	30.9 ± 2.0	55.8 ± 7.7	65.1 ± 3.6	49.9 ± 3.6	43.5 ± 12.6
Ge	32.8	195.6 ± 12.0^b^	43.1 ± 1.8^b^	324.1 ± 23.6^b^	< LoQ	< LoQ	465.3 ± 11.5^b^	< LoQ	< LoQ	91.5 ± 3.3^b^
	Content (g kg^−1^ DM)									
Si	0.1	9.8 ± 1.0^b^	18.9 ± 6.9^b^	20.3 ± 3.4^b^	0.8 ± 0.1^b^	0.5 ± 0.1^b^	12.3 ± 1.6^b^	16.7 ± 8.6^b^	5.6 ± 0.5^b^	7.8 ± 1.9^b^

Si contents ranged from 0.05 to 2% of DM while the sum of REEs concentrations were < 0.00012% in DM and Ge concentrations < 0.00005% in DM. Thus, Si supply from fenland vegetation demonstrated the highest potential among investigated strategic elements. The obtained concentrations were at least several orders of magnitude higher compared to both REEs and Ge. While cattail contained the lowest amount of Si, other species were characterized with much higher Si concentrations, with common reed and sedges being on top. The investigated fenland plant species revealed themselves as rather poor in Ge. Ge contents ranged from 43.1 (common reed) up to 465.3 µg kg^−1^ DM (reed canary grass). Ge was not detected or found in concentrations below quantification limit in cattail, sedges and rushes. Within REEs group, the highest concentrations were observed for Nd, Y, Gd and Eu. La and Ce were detected at a higher concentration, but only in one single sample and therefore are least reliable. None of the tested plants showed any presence of Tm and Lu. In the majority of the samples LREEs constituted more than half of total REEs, with the exception of two samples of common reed. Generally, the highest REEs content were found in broad-leafed cattail followed by common reed and the lowest in another sample of broad-leafed cattail.

Variation in the concentrations of individual elements were also noted within the same plant species. Among common reed samples harvested during summer but originating from different sites, the observed differences were relatively small for measured ∑HREE and Si concentrations compared to ∑LREE or Ge contents. Si concentration in common reed was two times lower for biomass harvested in winter when compared to biomass harvested in summer. Substantial dissimilarities in SE concentrations were also determined in broad-leafed cattail of different origin, which was especially evident in the concentrations of both ∑LREEs and ∑HREEs.

**Fig. 1:**
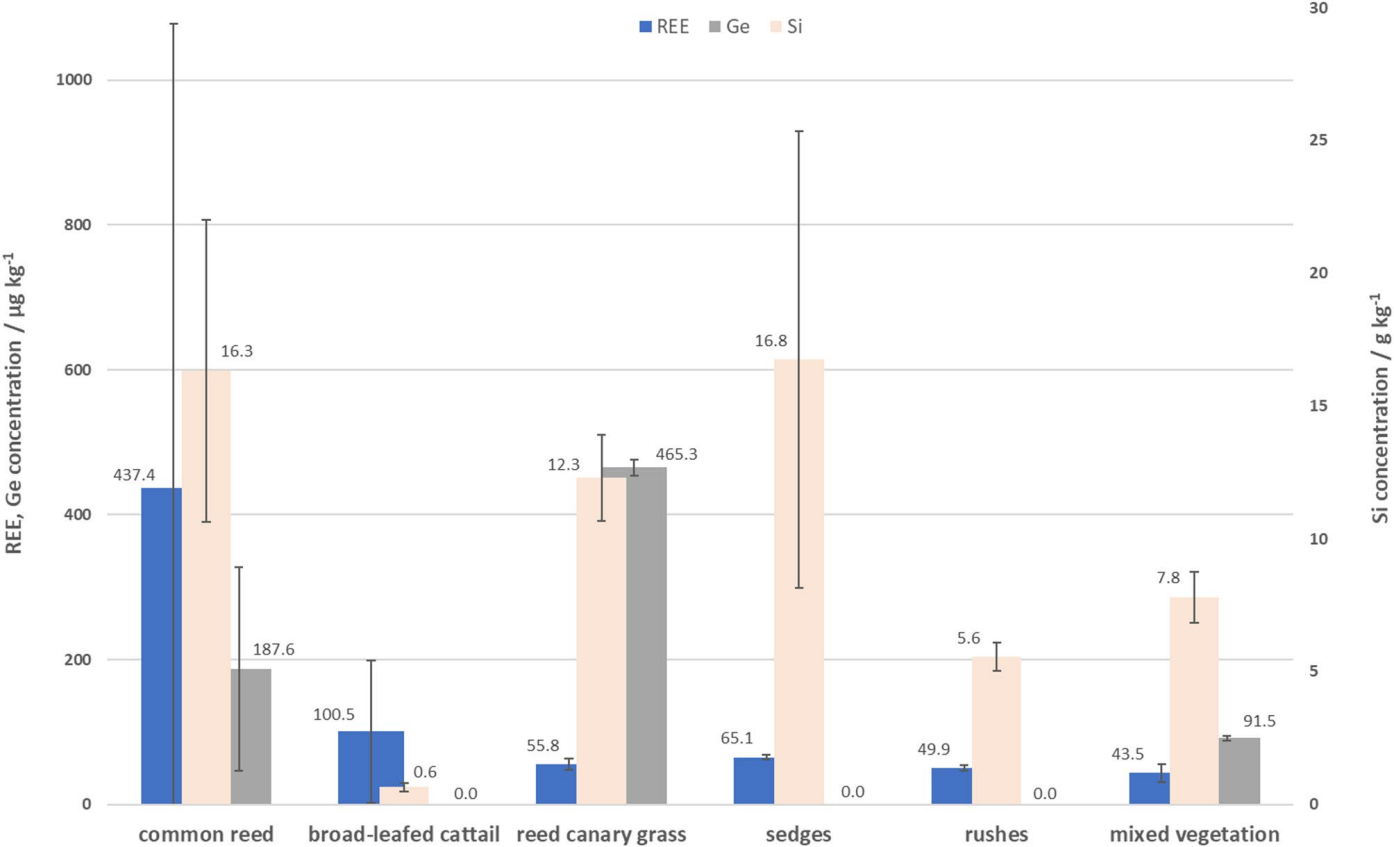
Mean concentrations of REEs, Ge and Si in fenland plant species. Due to the much higher Si concentrations, they are displayed on second (right) Y-axis, for which unit dimension is adjusted. Blue columns—REEs, grey columns—Ge, light orange columns—Si. Error bars represent standard deviation (SD).

It is important to point out the variation between obtained analytical results, noticed not only within the same species, but also within triplicates measured for individual samples, here displayed as standard deviation (SD). To identify the species with the highest potential for resource supply, concentrations determined within the same species (i.e. common reed and broad-leafed cattail) originating from different locations were averaged and the obtained mean values were further used to compare element contents between all species (Fig. 1). Sedges, common reed and reed canary grass contained the highest amount of Si. Reed canary grass showed considerably higher amounts of Ge than other species, having more than twice as much as in common reed and more than five times higher concentration than in mixed vegetation. Independent of the site of origin, common reed followed by cattail and sedges were the species with the highest amounts of REEs while mixed vegetation displayed the lowest concentration.

### Potential yields of strategic elements from rewetted fenlands

**Fig. 2:**
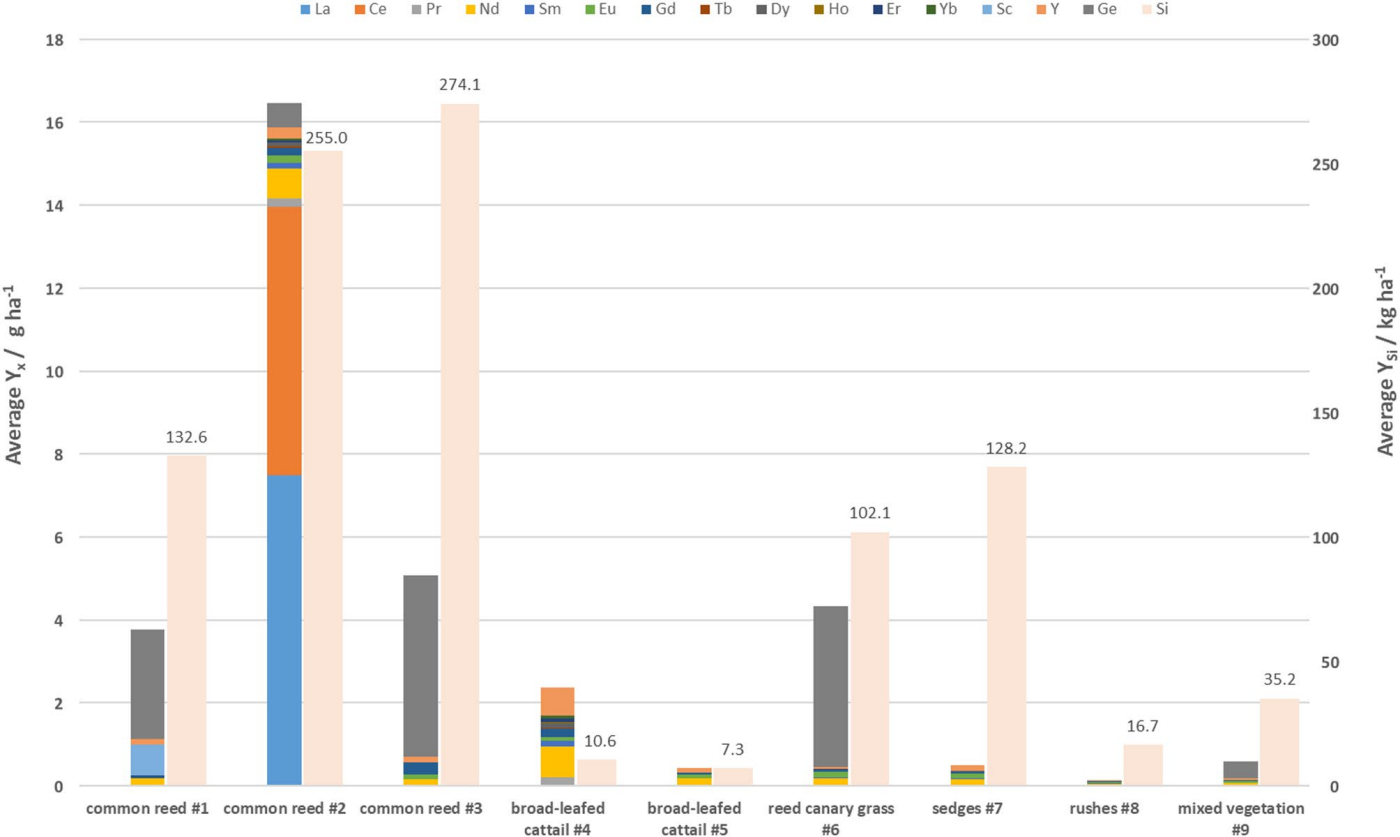
Average potential yields (Y) of individual elements (x) from fenland biomass samples. Due to the much higher Si yields, they are displayed on second (right) Y-axis, for which unit dimension is adjusted. Different colours represent individual elements.

The average element yields per hectare for all tested biomass samples are presented in Fig. 2**.**

Among all strategic elements investigated, the highest element yields were reported for Si. Si yields reached substantial levels in common reed samples with up to 274.1 kg Si ha^−1^. Sedges, reed canary grass and rushes achieved average yields from 16 to 128 kg Si ha^−1^ while Si yields were lowest in broad-leafed cattail samples with 7.3 kg Si ha^−1^. The average Ge yields ranged from 0.5 to 4.3 g Ge ha^−1^. Only little less (on average 3.8 g Ge ha^−1^) can be provided by reed canary grass, the species with almost the lowest biomass yield. For the majority of analysed samples, the potential for supplying REEs appeared to be rather low, with distinct differences observed between various plant species. Apart from common reed sample #2, for which both La and Ce yields correspond to almost 85% of the total REEs yield, other biomass types offered considerably lower yields of individual REEs, with values ranging from several dozen to several hundred mg ha^−1^, but still below 1 g ha^−1^. In total, the average REE yields ranged from 0.4 to up to 2.4 g ha^−1^ with both values found in broad-leafed cattail samples. Besides the high potential of common reed for La and Ce supply, obtained yields of Nd (broad-leafed cattail and common reed), Y (broad-leafed cattail) and Sc (common reed) could still be beneficial for supplying those elements. From these results it is also clear that biomass yield strongly influences the ultimate REEs output. For plant species with lower average biomass yields such as reed canary grass and sedges, respective REE yields were also notably lower. For rushes which are characterized by the lowest biomass yield, obtained REE yields were the lowest.

**Fig. 3:**
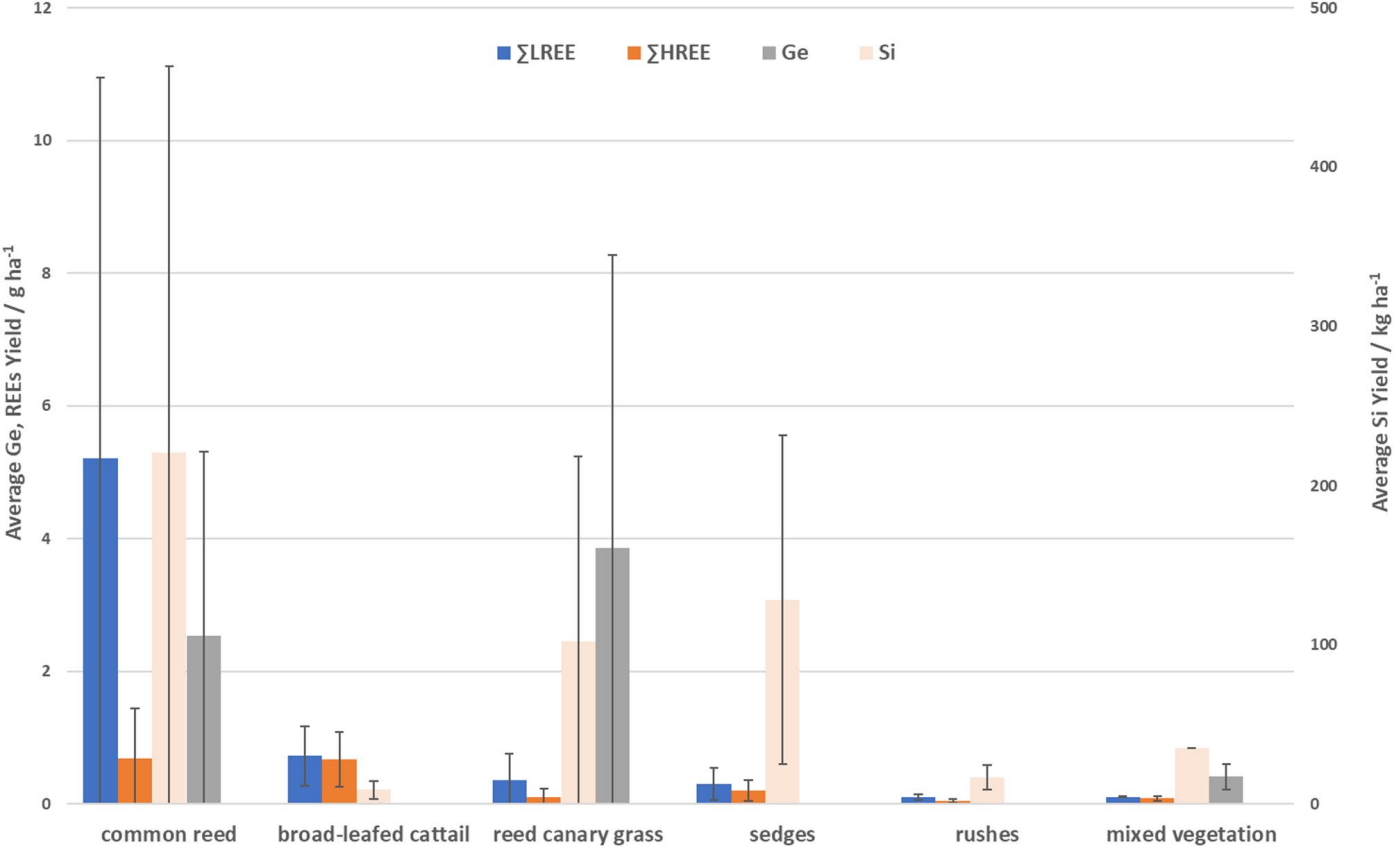
Average potential yields of REEs, Ge and Si for fenland plant species along with their standard deviation (SD). Due o the much higher Si yields, they are displayed on second (right) Y-axis, for which unit dimension is adjusted. Blue colour—∑LREEs, orange colour—∑HREEs, grey colour– Ge, light orange colour—Si. Error bars represent standard deviation (SD).

Figure 3 shows the mean element yields when aggregating all samples of the same species. The potential for Si supply was identified to be the highest for common reed, while reed canary grass showed the highest Ge accumulation per hectare. The REEs yield obtained from common reed were considerably higher than the yields obtained for other plant species. Broad-leafed cattail, sedges and rushes were less effective in terms of their REEs yield potentials, which for sedges was linked to the rather low biomass yields. Low biomass yields of rushes translated to very low yields of all strategic elements.

### Potential revenues

**Fig. 4:**
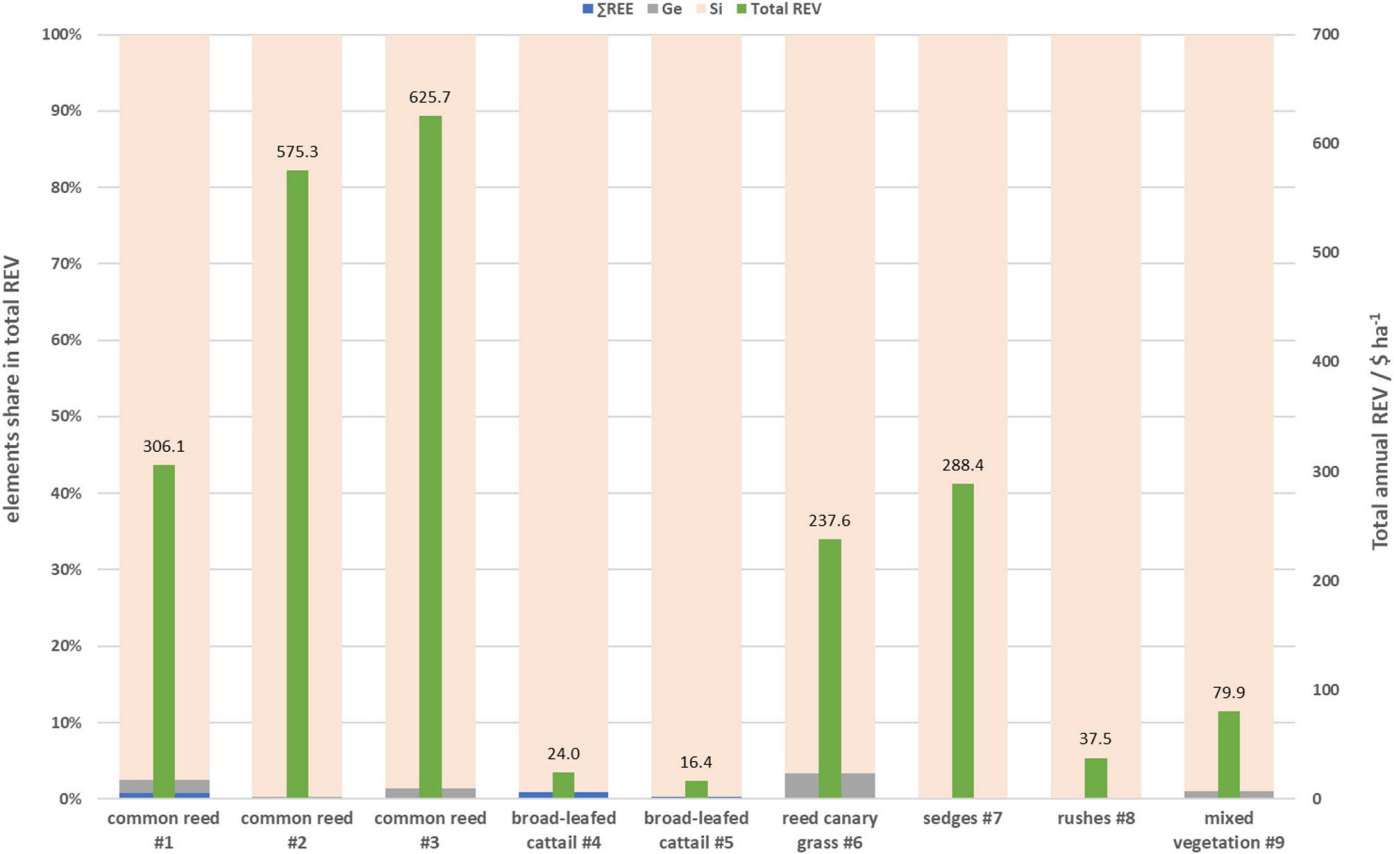
Annual average revenues (REV) and shares of REEs, Ge and Si acquisition from fenland plant samples. Blue colour—REEs, grey colour—Ge, light orange colour—Si and green colour—total average annual revenue.

The potential annual revenues generated solely from product (i.e. pure recovered element) sales (without extraction costs) differed considerably among investigated plant species (Fig. 4) as the element concentrations and yields varied. They ranged from 16 $ ha^−1^ for broad-leafed cattail to 625 $ ha^−1^ for common reed and they basically decreased in order: common reed > sedges > reed canary grass > rushes > broad-leafed cattail. Mixed vegetation offered several times higher revenue than broad-leafed cattail and rushes.

The highest revenues resulted from Si acquisition. This strategic element not only was accumulated by all tested fenland plant species, but also at very high levels. That translated to considerable shares in generated revenues, from 96.7 up to 99.9%. The revenues from Ge acquisition showed up to be substantially lower and ranged from 0.2 to 3.3% of total revenues generated. REEs share was negligible with maximum of 0.9%. In general, the highest share in revenues generated solely from REEs were derived from elements such as Eu, Nd, Dy and Tb (Fig. S1 in Supplementary Information).

**Fig. 5:**
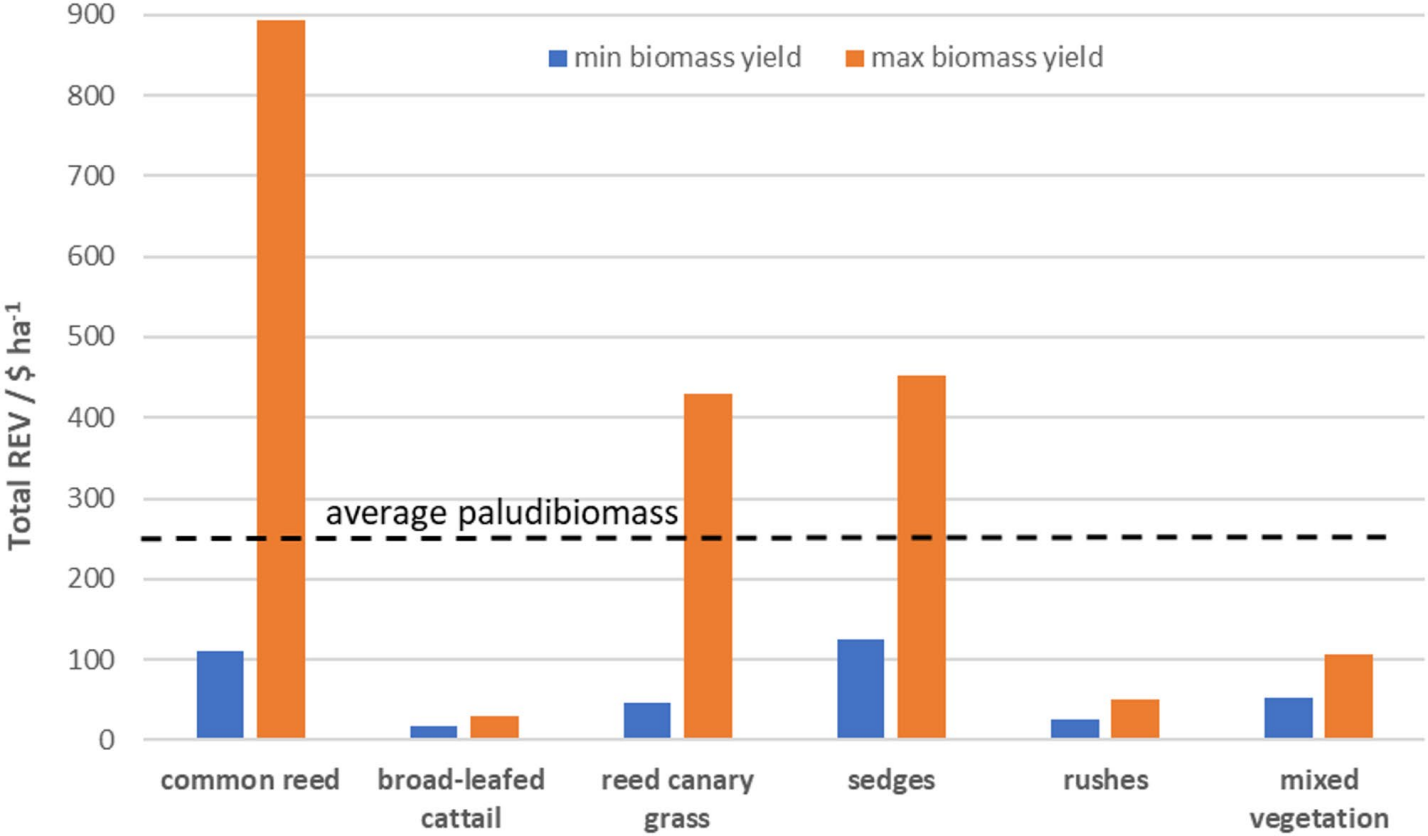
Total annual revenues (REV) for fenland plant species at minimum and maximum biomass yields. Blue colour—minimal biomass yield and orange colour—maximum biomass yield. Dashed line—total average annual revenue calculated for all fenland species tested.

Total revenues for all plant species investigated revealed remarkable differences between the plant species (Fig. 5). While expected total revenues from SEs from fenland biomass on average did not exceed 245 $ ha^−1^, potential average revenues could reach up to 502 $ ha^−1^ for common reed, 288 $ ha^−1^ for sedges and 237 $ ha^−1^ for reed canary grass. The exception was broad-leafed cattail, which would generate average total revenues of only 23.6 $ ha^−1^. This is lower than minimal outcomes obtained from the other species. Soft rushes could generate only slightly higher average revenues of 38 $ ha^−1^, twice less than mixed vegetation (80 $ ha^−1^). The individual shares in total potential revenues were 98.8% from Si, 1.1% from Ge and 0.1% from REEs.

## Discussion

Peatland soils are considered to be moderately rich in Si^63,68,69^ with Si enrichment being a consequence of the erosion of slopes and periodical flooding^70^. Core samples from boreal wetlands have been found to contain large amounts of amorphous silica^69^, also as determined for plants from the families *Cyperaceae* (sedges) and *Poaceae* (grasses)^61,71^. As such the plant samples from fenlands examined in this study should have a potential for Si accumulation. Indeed, Si concentrations obtained for common reed (*Poaceae*), reed canary grass (*Poaceae*) and sedges (*Cyperaceae*) were considerably high (16.3, 12.3 and 16.8 g Si kg^−1^ DM, respectively), accounting for up to 2% DM while Si contents found in rushes (*Juncaceae*) and broad-leafed cattail (*Typhaceae*) did not exceed 5.5 g kg^−1^ DM. Accordingly, the analysed fenland plant species can be classified as high (10 to 100 g Si kg^−1^ DM) or intermediate Si plant accumulators (5 and 10 g Si kg^−1^ DM)^42^. Their efficiency in Si accumulation is similar to other known high accumulators, such as rice (41.7 g Si kg^−1^ DM), wheat (24.6 g Si kg^−1^ DM) or barley (18.2 g Si kg^−1^ DM)^43,67^ (Table S1 in Supplementary Information).

Observed variations are assumed to result mainly from different capacities of plant roots to take up Si, with Si translocation to the shoot by transpirational volume flow through the xylem playing an important role^72–74^. Seasonal fluctuations within common reed were also noted between samples collected in winter and summer with Si concentration two times lower in winter season. Such fluctuations are likely due to the changes in the transpiration flux pattern along the growing season observed previously for reed stands in wetlands, with the highest transpiration rate determine in summer^75^. Reasons for such variation other than plant age can be assumed in site-conditions, large differences among genotypes within the same species^76^ or the phylogenetic position of the plant^71,77^.

Some of the factors affecting Si availability and accumulation in plants are likely to influence Ge uptake in a similar pattern. Its dependence on a variety of complex interactions of both soil-associated (e.g. absence of plant-available Ge) and plant-associated (e.g. inefficient uptake capabilities) factors has been demonstrated and discussed in several studies so far^44,60,78,79^. In our study, Ge was detected only in common reed and reed canary grass as well as in mixed vegetation, which suggests that Ge provision correlates strongly with the type of vegetation.

Comparing the obtained Ge concentrations with other studies was possible only for reed canary grass, which in our investigation turned out to be the most promising species for Ge recovery. Reed canary grass from fenland contained 465.3 µg Ge kg^−1^ DM, which is in the medium to upper range of Ge concentrations in reed canary grass cultivated on various mineral soils (591 µg Ge kg^−1^ DM^60^, 240 to 900 µg Ge kg^−1^ DM^80,81^). This finding contradicts other results, which indicate that soil organic matter binds Ge weaker than mineral phases^82^, but is in line with other studies^81,83^, which show organic matter content as relevant binding factor for sites with slower mineralization due to high water levels.

Regardless, compared to cultivated plants, e.g. maize (84 µg Ge kg^−1^ DM^74^ and 360 µg Ge kg^−1^ DM^79^) or sunflower (21 µg Ge kg^−1^ DM^74^), the Ge concentrations found in reed canary grass were considerably higher, which further demonstrates the potential of paludibiomass to accumulate this element.

The concentrations of REEs in fenland samples turned out to be rather low. With 30.9 to 1176.6 µg kg^−1^ DM they are several orders of magnitude lower than those detected in well-known REEs plant hyperaccumulators (e.g. 760.7 mg kg^−1^ DM for *Dicranopteris dichotoma,* 593.4 mg kg^−1^ DM for *Cyperus rotundus* and 514 mg kg^−1^ DM for *Phytolacca Americana*^84^) and clearly lower than REEs contents in some cultivated crops (7400 µg kg^−1^ DM for maize (*Zea mays *L.) or 1916 µg kg^−1^ DM for wheat (*Triticum aestivum*)^65,66^). The reported differences most likely result from both the primary availability of REEs in soils as well as environmental conditions such as redox potential and pH, which are considered to be main factors controlling the mobility of REEs^51,85,86^. The mechanism of this mobilisation is explained by the dissolution of Fe–Mn oxides under reducing conditions, which leads to the subsequent release of adsorbed REEs^85^.

Direct comparison of REEs concentrations in paludibiomass with literature data was not possible. The only data available is for reed canary grass in Germany^79,83^ and soft rushes in Poland^87^, which did not originate from organic but from mineral soils. Generally, element contents were lower in the fenland plant species. For instance, our results for reed canary grass growing on organic soil (i.e. 55.8 µg REEs kg^−1^ DM, sample #6) are 10 times lower than the REEs concentration found in mining areas of Freiberg, Germany (524 µg REEs kg^−1^ DM)^83^. The differences can be noticed also for individual elements found in reed canary grass. Similarly, our results obtained for rushes (49.9 µg REEs kg^−1^ DM) are in the lower range of concentrations found in rushes harvested on 15 mineral sites in Poland (28 to 2700 µg REEs kg^−1^ DM)^87^. Individual capabilities of plants to acquire REEs from soil differ, which is reflected in our results with common reed exceeding the contents of the other tested fenland plant species. The growth stage of the plant could also play an important role, as the REE concentration patterns are a function of time^59^. However, the question of whether this feature dominates or is overlapped by other biotic and abiotic factors remains open.

Based on these first insights on SE concentrations in fenland plant species, reed canary grass, common reed and sedges can be considered as offering the highest potentials particularly for Si and Ge. Reported higher affinity toward accumulation of those two elements might result directly from their biogeochemical similarities as well as shared uptake and transport system in plants, yet the exact mechanism underlying Gi/Si translocation and its limitations has not been fully elucidated^60,88,89^. A variety of soil- and plant associated factors as well as hydrological conditions and harvesting time should be further investigated to maximize the efficiency of SEs supply from fenland vegetation. SE distribution patterns in aerial plant organs and roots should also be taken into account in the assessment of supply potential^53,54,59^.

The reported differences in SE concentrations between fenland species resulted in varying potential revenues. In addition to the market price and the SEs content in plant material, the respective biomass yield was the key factor for the ultimate total revenue. For instance, while average Si contents in sedges and common reed biomass were similar (16.8 and 16.3 g Si kg^−1^ DM), the calculated Si-based revenues were more than 70% higher for common reed, which is characterized by 75% higher average annual biomass yield in comparison to sedges (13.5 and 7.6 t DM ha^−1^). Despite high Ge market prices and higher contents in plants compared with REEs, the potential revenues could reach up to maximum of only 8 $ ha^−1^ for reed canary grass. Ge concentrations in fenland species did not exceed 0.5 mg Ge kg^−1^ DM, while some studies suggest economic usability of Ge for concentration above 10 mg kg^−1^ of DM^90,91^. Lower concentrations could be acceptable only if other elements were separated^90^. REEs accounted for only 0.1% of total revenues. Minimal REEs concentration for remunerative REEs acquisition from plants should be in a range from 3 to 30 mg kg^−1^ DM^92^. This is significantly more than concentrations determined in our study, among which only one sample contained more than 1 mg of REEs per kg of DM.

With Si and Ge accounting together for more than 99.8% of the calculated total revenues, simultaneous acquisition of these two elements seems to be promising. Such a dual approach might be reasonable due to geochemical similarities of both elements and thus their common presence together in soils and plants in form of phytoliths^60,92^. The recent discovery that these small chemical structures are also enriched with REEs, which makes them a very attractive target for further SEs extraction^93,94^, may also be of great importance here. Nonetheless, the separation processes for Si and Ge or other elements together with their costs need to be taken into account and further optimized.

Both the dedicated cultivation of crops and the multi-step extraction of strategic elements are associated with high costs^91^ whereas the potential revenues generated from SEs from typical fenland species could be on average between 237 to 502 $ ha^−1^ for the most promising plant species. However, the integration into existing process chains might hold the potential to considerably reduce costs for element extraction, while maximising revenue for paludibiomass as a whole.

Current and future biomass preservation and pretreatment processes could support dissolving of desired elements out of the plant matrix. As a commonly applied process for preservation and storage of herbaceous biomass, ensiling leads to a significant decrease in the pH value of the compacted and airtight sealed plant material by microbial conversion of easily soluble carbohydrates to high amounts of various short-chain organic acids^95–97^. So far, little is known about ensiling characteristics and process of paludibiomass and the potential to promote element leaching. Drawing from previous experiences for a better degradation of lignocellulosic biomass in biogas production^98–100^, also fungal pretreatment could be a promising option to accumulate SEs in fungal fruiting bodies or mycelium and subsequently extract them. Screening for other microorganisms and/or consortia should also be considered as an alternative^101^.

The extraction of strategic elements as co-products from biomass conversion processes for material or energy use might offer potential sources of obtaining additional revenues within existing paludibiomass utilization pathways. Strategic elements acquisition and extraction could be integrated into multi-output value networks, post-processing and consumption residues, for sustainable use of paludibiomass thus increasing economic viability. With a variety of functional materials such as paper products or packaging, insulation or building materials or peat substitutes in the core product portfolio, the use of sides-streams can flexibly be adjusted to regional capabilities for bioenergy production, drawing from appropriate individual processing steps and complementing the material cycle.

At the current state of art, above all anaerobic digestion could offer a technologically and economically feasible potential to dissolve strategic elements out of the biomass matrix^102^. So far it has been shown that strategic elements occur in higher concentrations in digestates than in their plant feedstocks^103,104^, whereat this enrichment can be explained by the degradation of organic matter and transfer of carbon to the biogas during the anaerobic digestion process. With regard to a multifunctional use and maximum valorisation of paludibiomass, studies on residue characterisation, their utilisation as feedstock in biogas reactors and the fate of strategic elements during the digestion process are needed. The challenge will be the recovery of strategic elements from the digestates. Compared to classical approaches using bioleaching via specific acid production of pure cultures in a separate fermentation step to recover strategic elements from ore deposits, soils, electrical waste or wastewater sludge^101,105,106^, the usage of highly diverse anaerobic microbiomes specialized for hydrolysis and particularly acidification seems to be promising for leaching embedded high-value resources from digestates^107^. One option could be an on-demand adaptable side-stream with enriched microbiomes producing a multitude of organic acids to integrate a bioleaching system for the extraction of SEs from digestates.

The environmental costs of SEs acquisition from peatlands, such as possible Si depletion from peatland soils, cannot be ignored, as it poses the threat for ecosystem stability. Therefore, an appropriate assessment should be carried out in parallel with other studies.

In summary, the results of this study on SE concentrations in fenland plant species indicate research demand on species-, site- and season-specific element contents and improved valorisation through the integration of element extraction into harvesting, conserving (in terms of extending storage time) and processing chains for paludibiomass as part of sustainable bioeconomy.

## Conclusions

This study provides the first essential information on concentrations of SE in paludibiomass. Reported substantial differences among SEs concentration in plant species point out the necessity for further studies to fully explore the topic.

This study indicates that Si and Ge are most promising for effective and efficient recovery. From the initial estimation of potential revenue, the application of paludiculture focused solely on SE recovery is not recommend. Instead SE extraction should be integrated into existing process chains and thus complement material valorisation.

The presented investigation also reveals existing research gaps, which need to be filled in the future. Key point will be to determine the exact correlations between environmental factors (such as pH, water level, redox potential) and the accumulation of elements in plant tissues. The possibility of phytholiths serving as SEs reservoirs appears to be very promising direction for future studies. Seasonal fluctuations in SE accumulation as well as distribution patterns should also be identified.

## Materials and methods

### Biomass samples

Biomass samples were taken from various rewetted fields in the fenland-rich federal states of Mecklenburg-Western Pomerania and Brandenburg in northeast Germany (Table 2). Chosen fields were intended to reflect the spectrum of vegetation that typically establishes after rewetting of fen peatlands^20^. Fields at the sampling sites were harvested within the common practical management for grass land by the farmers during the usual harvest periods and with the available farm machinery. After mowing and field drying the biomass was picked up and compacted with large round balers or small square balers. Hay bales from patches with homogeneous vegetation were chosen for investigation, transported to the research facility and stored in a hall.

**Table 2:** Origin of biomass samples. The average biomass yields based on fenlands literature data (references in brackets).

Sample No	Vegetation		Location		Harvest period	Average biomass yield
	Common name	Latin name	Federal state	Site name		t DM ha^−1^ a^−1^
1	Common Reed	*Phragmites australis*	Mecklenburg-Western Pomerania	Kamp	Winter 2020	13.5^90^
2	Common Reed	*Phragmites australis*	Mecklenburg-Western Pomerania	Binsenberg	Summer 2020	
3	Common Reed	*Phragmites australis*	Brandenburg	Gollwitz	Summer 2020	
4	Broad-leafed Cattail	*Typha latifolia *L.	Mecklenburg-Western Pomerania	Kamp	Winter 2020	13.9^108–110^
5	Broad-leafed Cattail	*Typha latifolia* L.	Mecklenburg-Western Pomerania	Neukalen	Winter 2021	
6	Reed Canary Grass	*Phalaris arundinacea* L.	Brandenburg	Kremmen	Summer 2020	8.3^90,110^
7	Sedges	*Carex* spec	Mecklenburg-Western Pomerania	Binsenberg	Summer 2020	7.7^111^
8	Rushes	*Juncus* spec	Mecklenburg-Western Pomerania	Binsenberg	Summer 2020	3.0^112^
9	Mixed vegetation	–	Brandenburg	Gollwitz	Summer 2021	4.5^90^

### Chemical analyses

#### Sample preparation

The crushed sample material was first dried at 105 °C until constant mass. From this, 3 subsamples of approx. 0.1 g each were weighed and decomposed with an acid digestion method for the determination of the rare earth metals and 2 subsamples (lower number due to limited number of available crucibles) were weighed and decomposed with a basic digestion method for the determination of silicon and germanium.

The acid digestion was carried out in quartz vessels with the addition of 2 mL of concentrated HNO_3_ (65% (*v/v*)) in a High Pressure Asher (HPA, Anton Paar, Ostfildern-Scharnhausen, Germany) at 300 °C for 3 h. The solution was then quantitatively analysed. The solution was then quantitatively transferred to a 15 mL centrifuge tube and made up to 15 mL. A 1:10 dilution was prepared from this. This was provided with yttrium as an internal standard (2.5 µg L^−1^ yttrium in the measuring solution). Another 1:10 dilution was prepared from a partial sample of sample no. 9, which was provided with a multi-element standard (1 µg L^−1^ analyte concentration in the measuring solution). The measurement signals of this spiked sub-sample and the non-spiked sub-sample of sample no. 9 served as a matrix-adjusted calibration for all acid-digested samples.

The basic digestion began with the ashing of the samples in silver crucibles for 3 × 1 h at 500 °C in a microwave oven (MLS Start, Leutkirch im Allgäu, Germany). Subsequently, approx. 400 mg sodium hydroxide was added to each sample and fusion digestion was carried out at 600 °C for 2 h in the same microwave oven. After cooling, the crucibles were half filled with Milli-Q water and left overnight. The contents of the crucible were quantitatively transferred to 50 mL centrifuge tubes, which were filled to approx. 45 mL, 750 µL HNO_3_ (65% (*v/v*)) was added and filled to 50 mL. These samples were then divided in half into 2 subsamples each. Yttrium was added to both subsamples as an internal standard (1 µg L^−1^ yttrium in the test solution). One of the two subsamples was additionally spiked with a silicon standard (5 mg L^−1^ silicon concentration in the test sample) and a germanium standard (1 µg L^−1^ germanium concentration in the test sample). Finally, both subsamples were made up to 50 mL. The measurement signals of these spiked subsamples and the undoped subsamples were used separately for each melt digestion as a matrix-adjusted calibration.

#### Sample analysis

The sample solutions were analysed on a high-resolution, sector field ICP-MS (Element 2, Thermo Fisher Scientific, Dreieich, Germany).

A self-aspirating glass nebulizer from Micromist with an aspiration rate of 200 µL min^−1^, a glass cyclone nebulizer chamber, a quartz injector tube and nickel cones were used for the acid-digested samples. The basic digested samples were analysed with a self-aspirating nebulizer made of PFA with an aspiration rate of 100 µL min^−1^, a PFA nebulizer chamber, a sapphire injector tube and nickel cones.

Scandium and Yttrium analysis was carried out with a Triple-quadrupole ICP-MS (ICP-QQQ-MS, Agilent 8900, Waldbronn, Germany). Indium was used as internal standard. To measure Scandium and Yttrium, a 1:10 dilution of the acidic digestion solution was prepared again and Indium was added as internal standard at a concentration of 5 μg L^−1^. Scandium was analysed in the “H_2_ mode”, while Yttrium results are merged from “no gas, H_2_ and He mode”.

### Potential yields and revenues of strategic elements from fenlands

In this publication, the commonly used term “phytomining” is deliberately replaced by the terms “acquisition” and “supply” to distinguish between the origin of the strategic elements, which in this case is limited to organic (i.e. peatland) soils. In general, several factors influence the economic viability of SEs acquisition from plants. Biomass yield, SE concentrations in plants and market prices of individual elements are of major importance. The values of the two first parameters are necessary for calculation the potential yield of SEs per hectare. For the calculations of potential yields of SEs from fenlands literature data concerning average biomass yields for investigated vegetation types was used. These annual values were as follows: from 3 to 24 t DM ha^−1^ for common reed^90^, from 7.8 to 20 t DM ha^−1^ for broad-leafed cattail^108–110^, from 1.6 to 15 t DM ha^−1^ for reed canary grass^90,110^, from 3.3 to 12 t DM ha^−1^ for sedges^111^, from 2 to 4 t DM ha^−1^ for rushes^112^ and from 3 to 6 t DM ha^−1^ for mixed vegetation^90^. Individual SE yields Y_X_ were calculated according to Eq. (1):

1 $$\begin{array}{c}{Y}_{X}={B}_{Y }\cdot {X}_{c}\end{array}$$

where Y_X_ refers to the yield of individual element X (kg ha^−1^), B_Y_ refers to biomass yield of individual plant species, here given as common reed, broad-leafed cattail etc. (kg DM ha^−1^) and X_C_ is the determined concentration of individual element X in the plant species (kg kg^−1^ DM). When possible, both minimal and maximal SEs yields were calculated.

To assess the potential revenues generated from product (i.e. pure recovered element) sale, recent market prices of SEs were considered. Due to the lack of data regarding both the effectiveness of enrichment as well as the costs of extraction processes, more sophisticated, full economic analysis could not be performed and was limited to a very basic calculation. Up to the time of publication, there is no reliable source that would provide prices for all strategic elements mentioned in this work for the specified time period. As such the calculated revenues are based on average individual SEs prices from 2020^92^ with the exception for Si, for which the market price from 2024 was used^113^. In 2020 REEs prices ranged from 4.3 $ kg^−1^ for La, through 74.9 for Nd, 681.2 for Lu, and reached up to 3211.1 $ kg^−1^ for Sc. While Si is nowadays cheaper than any REE (2.3 $ kg^−1^), Ge price (2045.1 $ kg^−1^) significantly exceeded the prices of most other elements. Calculations were omitted for Tm since this element was not detected in any sample and market prices were not available. The theoretical revenue potential for recovering individual elements was calculated according to Eq. (2):

2 $${REV}_{X}={Y}_{X}\cdot {P}_{X}$$

where REV_X_ refers to the economic value of recovery of individual element X ($ USD ha^−1^), Y_X_ refers to the yield of individual element X (kg ha^−1^), and P_X_ is the market price of individual element X in the sample ($ USD kg^−1^).

### Statistical analysis

Quantitative analyses were limited due to the fact that around a half of the analytical measurements were below respective limits of quantification. Presented concentrations of individual as well as total amounts of SEs are therefore probably underestimated. In most cases the mean values resulting from either double or triple measurements of element concentrations are given. Otherwise, the result of the single measurement is shown. The variation between measurements with respect to the individual element concentration for each plant sample as well as for sum of REEs for both individual plant species and between the same plant’s species collected at different sites are represented by standard deviation (SD).

## Electronic supplementary material

Below is the link to the electronic supplementary material.

Supplementary Material 1

## Data Availability

All data generated or analysed during this study are included in this published article and Supplementary Information file.
