# Supplementary material for: Potentials of typical plant species from rewetted fenlands for the supply of strategic elements
Source: Sci Rep. 2025 Jun 20;15:20149. doi: 10.1038/s41598-025-05180-0 (PMC12181310; doi:10.1038/s41598-025-05180-0)
Supplement: Supplementary file 1 — Supplementary Material 1 [file 41598_2025_5180_MOESM1_ESM.pdf]

Potentials of typical plant species from rewetted fenlands for the supply of strategic elements

Karina Michalska[1*], Monika Heiermann[1], Carsten Lühr[1], Björn Meermann[3], Ralf Pecenka[1], Andreas Schulz[3], Nicole Langhammer[3], Susanne Theuerl[1], Annette Prochnow[1,2]

[1] Leibniz Institute for Agricultural Engineering and Bioeconomy (ATB), Max-Eyth-Allee 100, 14469 Potsdam, Germany
[2] Albrecht Daniel Thaer Institute for Agricultural and Horticultural Sciences, Faculty of Life Sciences, Humboldt-University of Berlin, Hannoversche Str. 27, 10115 Berlin, Germany
[3] Federal Institute for Materials Research and Testing (BAM), Division 1.1 – Inorganic Trace Analysis, Richard-Willstätter-Straße 11, 12489 Berlin

* Corresponding author
kmichalska@atb-potsdam.de
phone: +49 331 5699-216

Keywords: peatlands, paludiculture, Ge, Si, rare earth elements, bioeconomy

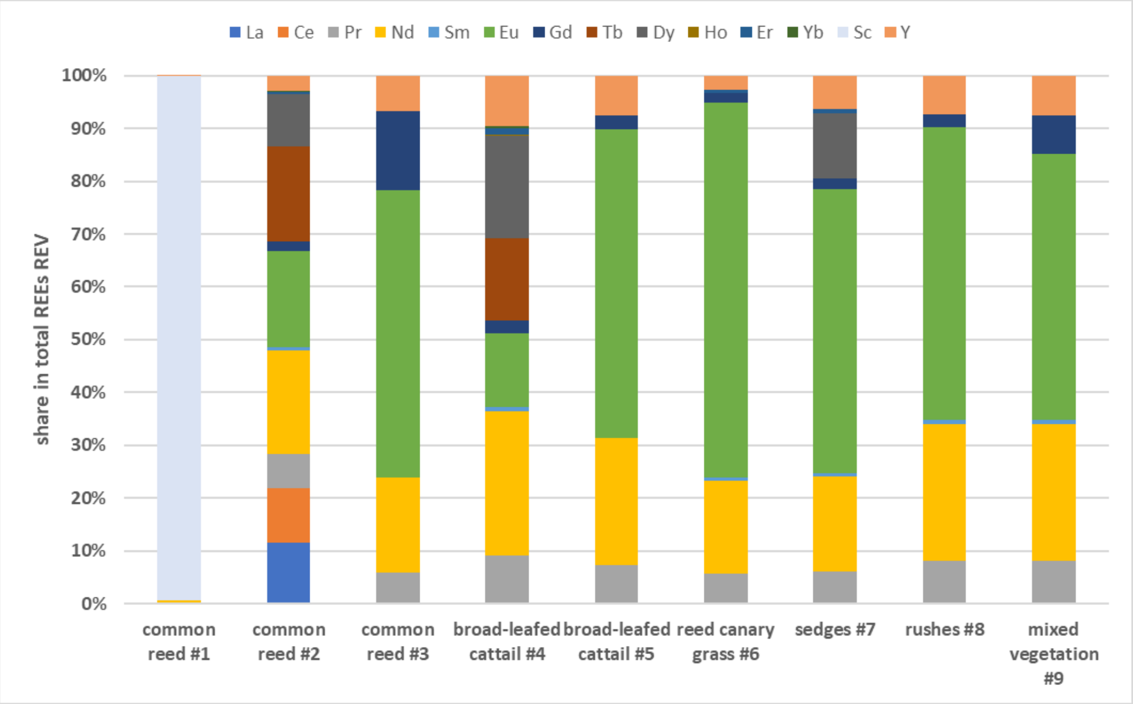

Figure S 1 Individual elements' shares in total REEs revenues (REEs REV) from fenland plant samples. Different colours represent individual elements.

Table S 1 Individual concentration of strategic elements in reed canary grass, maize and rice. n.d. – not determined.

	**Concentration / µg kg^{-1} DM (for Si g kg^{-1} DM)**			
Element	Reed canary grass	Reed canary grass	Maize	Rice
Source	This study	REEs [1]<br>Si, Ge [2]	REEs [3]<br>Si, Ge [4]	REEs [3]<br>Si, Ge [4]
Sc	< 49,6	3 – 328	n.d.	n.d.
Y	6.6	6 – 524	15.2 - 150.5	16.0 – 282.0
La	< 339.4	10 – 887	18.9 - 342.1	20.9– 372.4
Ce	< 286.5	20 – 1824	34.4 - 632.9	34.9 – 716.0
Pr	4.4	2 – 203	4.1 - 73.0	6.3 – 84.6
Nd	16.6	8 – 835	16.2 - 249.5	13.5 – 288.0
Sm	3.9	6 – 162	4.3 - 49.1	3.8 – 50.4
Eu	17.6	1 – 60	1.1 – 8.6	1.9 – 15.9
Gd	4.9	2 – 135	3.3 – 52.1	7.1 – 49.8
Tb	< 1.7	< 1 – 53	< 1.6 – 6.5	1.2 – 9.5
Dy	2.6	3 – 123	2.6 – 46.8	4.1 – 54.7
Ho	< 1.8	< 1 – 65	< 2.1 – 9.7	0.7 – 10.9
Er	1.9	< 1 – 96	1.4 – 21.9	2.3 – 33.5
Tm	< 1.8	< 1 – 67	< 1.1 – 3.0	< 2.9 – 4.9
Yb	< 2.0	0.5 – 74	2.0 – 17.9	1.6 – 46.3
Lu	< 2.4	< 0.5 – 59	< 0.8 – 3.2	< 1.7 – 5.9
Ge	465.3	666	84	170
Si	12.3	3.2	1.900	2.900

References

1. Sager, M. & Wiche, O. Rare earth elements (REE): Origins, dispersion, and environmental implications—A comprehensive review. *Environments*. **11**(2), 24 (2024). doi: 10.3390/environments11020024
2. Kaiser, S., Wagner, S., Moschner, C., Funke, C. & Wiche, O. Accumulation of germanium (Ge) in plant tissues of grasses is not solely driven by its incorporation in phytoliths. *Biogeochemistry*. **148**(1), 49-68 (2020). doi: 10.1007/s10533-020-00646-x
3. Li, F .L., Shan, X. Q., Zhang, T. H. & Zhang, S. Z. Evaluation of plant availability of rare earth elements in soils by chemical fractionation and multiple regression analysis. *Environ Pollut*. **102**(2-3), 269-277 (1998). doi: 10.1016/S0269-7491(98)00063-3
4. Okoroafor, P. U., Ogunkunle, C. O., Heilmeier, H. & Wiche, O. Phytoaccumulation potential of nine plant species for selected nutrients, rare earth elements (REEs), germanium (Ge), and potentially toxic elements (PTEs) in soil. *Int J Phytoremediation*. **24**(12), 1310-1320 (2022). doi: 10.1080/15226514.2021.2025207
